# A Neuronal Cell Line Model for Studying Camel Prions

**DOI:** 10.3390/pathogens15050472

**Published:** 2026-04-27

**Authors:** Basant Abdulrahman, Shabboo Rahimi Aqdam, Matteo Mosca, Hanaa Ahmed-Hassan, Melissa Razcon-Echeagaray, Lia Popa, Sabine Gilch, Baaissa Babelhadj, Gabriele Vaccari, Hermann M. Schätzl

**Affiliations:** 1 Calgary Prion Research Unit, University of Calgary, Calgary, AB T2N 4Z6, Canada; baabdulr@ucalgary.ca (B.A.); shabboo.rahimiaqdam@ucalgary.ca (S.R.A.); matteo.mosca@ucalgary.ca (M.M.); hanaasalahahmed.hass@ucalgary.ca (H.A.-H.); melissa.razconecheag@ucalgary.ca (M.R.-E.); iuliana.popa@ucalgary.ca (L.P.); sgilch@ucalgary.ca (S.G.); 2 Faculty of Veterinary Medicine, University of Calgary, Calgary, AB T2N 4Z6, Canada; 3 Hotchkiss Brain Institute, Cumming School of Medicine, Calgary, AB T2N 4Z6, Canada; 4 Snyder Institute for Chronic Diseases, Cumming School of Medicine, Calgary, AB T2N 4Z6, Canada; 5 Department of Biochemistry and Molecular Biology, Faculty of Pharmacy, Helwan University, Cairo 11795, Egypt; 6 Zoonoses Department, Faculty of Veterinary Medicine, Cairo University, Giza 12211, Egypt; 7 Department of Natural Sciences, Ecole Normale Supérieure de Ouargla, Cité Ennasr, Ouargla 30000, Algeria; babelhadjbaaissa@gmail.com; 8 Istituto Superiore di Sanità, Department of Food Safety, Nutrition and Veterinary Public Health, 00161 Rome, Italy; gabriele.vaccari@iss.it

**Keywords:** prion, prion disease, camel prion disease, prion infection, cell culture models, CAD5 cells, gene-editing, CRISPR-Cas/9, knock-out cells

## Abstract

Prion diseases are fatal neurodegenerative disorders that affect humans and animals, caused by the conformational conversion of the normal cellular prion protein (PrP^C^) into its misfolded, infectious isoform PrP^Sc^. Recently, camel prion disease (CPrD) was identified in dromedary camels (*Camelus dromedarius*) in Algeria. Due to the potential implications for animal and human health, as well as the possible socio-economic impact in Mediterranean regions where camels play a pivotal role as a source of food, in-depth characterization of camel prions is important to increase our understanding of camel prion disease. We developed a neuronal cell line model for studying the molecular features of camel prion infection. We genetically edited mouse neuronal CAD5 cells to generate CAD5 PrP knockout (KO) cells. We then used lentiviral transduction to generate CAD5 cells expressing camel PrP (CAD5-camel-PrP). Following infection of these cells with a CPrD-positive camel brain homogenate, we observed PrP^Sc^ signals at various passages, as indicated by immunoblotting analysis. RT-QuIC (Real-Time Quaking-Induced Conversion) assays further supported these findings, demonstrating transient prion conversion activity in the CPrD-infected CAD5-camel-PrP cells. Taken together, our data describe the first neuronal cell line permissive to camel prion infection, a novel in vitro tool for mechanistic studies of camel prion disease.

## 1. Introduction

Prion diseases, or Transmissible Spongiform Encephalopathies (TSEs), are fatal infectious neurodegenerative disorders affecting humans and other mammals. Prions are “proteinaceous infectious particles” that undergo conformational changes to induce the production of stable aggregated structures and initiate prion disease [1,2]. Prions possess self-propagating capabilities, as they can catalyze a profound conformational change from cellular prion protein PrP^C^ to an aggregation-prone structure, ultimately leading to the accumulation of misfolded and aggregated infectious PrP^Sc^ in the brain [3]. Cellular PrP^C^ is non-infectious, typically monomeric, and protease-sensitive. In contrast, PrP^Sc^ is infectious, aggregated, and partly resistant to proteolytic digestion by the proteinase K (PK) enzyme [4]. Examples of human prion diseases are Creutzfeldt-Jakob disease (CJD), Gerstmann–Straussler–Scheinker syndrome (GSS), fatal familial insomnia (FFI), and kuru [5]. Animal prion diseases comprise bovine spongiform encephalopathy (BSE) or mad cow disease in cattle, scrapie in sheep and goats, and chronic wasting disease (CWD) in deer and elk [6,7,8]. Prion diseases are prototypical protein misfolding diseases. Prion-like mechanisms, involving transmission of aggregates of misfolded proteins from cell to cell, were reported in various human neurodegenerative diseases, including Huntington’s disease [9], amyotrophic lateral sclerosis (ALS) [10], and Alzheimer’s and Parkinson’s diseases [11,12,13,14].

In 2018, Camel Prion Disease (CPrD) was reported in farmed dromedary camels during an antemortem slaughterhouse inspection in Ouargla, Algeria. In that study, 3.1% of inspected camels exhibited clinical signs of the disease [15], and later in Tunisia [16]. Camels with CPrD exhibit progressive symptoms, including weight loss, aggressiveness, ataxia of hind limbs, hypersalivation, teeth grinding, excessive nervousness, typical down and upward head movements, falls and difficulty getting up. Pathologically, CPrD presents with spongiform changes and the accumulation of PrP^Sc^, hallmarks of prion diseases. The clinical progression of CPrD covers weeks to months, and the disease is always fatal [15]. The origin of CPrD is unclear, but it is unlikely to be caused by inherited genetic mutations, as studies show no differences in the PRNP gene between CPrD-positive and unaffected camels [17]. While the zoonotic potential of CPrD remains uncertain, the widespread consumption of camel products, such as meat and milk, in Africa highlights the need to assess its interspecies transmission risk. The neurological symptoms of CPrD have been observed in adult dromedaries, and reports from breeders and slaughterhouse personnel have suggested its presence in dromedary populations since the 1980s [15]. This concern is amplified by comparisons to BSE, which crossed the species barrier and caused variant Creutzfeldt-Jakob disease (vCJD) in humans [18,19]. Investigating CPrD’s biology is crucial for public health, livestock and industry management. To our knowledge, no cell culture model currently exists to study camel prion disease, presenting a major gap in prion research. Developing such models would enable studies on CPrD prion propagation, molecular mechanisms, and cross-species transmission.

Recent advances in genetic engineering have enabled modifications of the host PrP gene to better match incoming prion strains. The application of gene-edited cell lines and transgenic animal models bridges the gap caused by the species barrier, offering platforms to study prion propagation across diverse hosts. In cell culture models, the species barrier phenomenon can lead to inefficient prion infection or no infection at all. Our study utilized CRISPR-Cas9 to achieve a complete KO of endogenous PrP in CAD5 cells. This approach ensures a neutral cellular background, allowing efficient transduction with species-specific PrP and facilitating propagation of prions otherwise not replicating in such mouse cells [20,21].

In this study, we decided to develop a neuronal cell line model for studying the molecular features of camel prion infection. We used CAD5 PrP-knockout cells and lentiviral transduction to generate CAD5 cells stably expressing camel PrP (CAD5-camel-PrP). Upon infecting CAD5-camel-PrP cells with a CPrD-positive camel brain homogenate, we observed PrP^Sc^ signals at various passages as indicated by Western blot analysis. RT-QuIC (Real-Time Quaking-Induced Conversion) assays further supported these findings, demonstrating prion conversion activity in CAD5-camel-PrP cells infected with CPrD. In addition, we generated CAD5 cells stably expressing bank vole PrP (CAD5-bank vole-PrP) to investigate the role of bank vole PrP as a universal prion acceptor. When we challenged the CAD5-bank vole-PrP cells with CPrD-positive camel brain homogenate, these cells did not propagate CPrD prions, as confirmed by immunoblotting and RT-QuIC results. Taken together, our data describe the first neuronal cell line susceptible to camel prion infection, offering a novel in vitro tool for mechanistic studies of camel prion disease.

## 2. Materials and Methods

### 2.1. Reagents

Proteinase K (PK) (Roche, South San Francisco, CA, USA; 03115879001), Pefabloc inhibitor (Roche, 11286700). Immunoblotting was done using the enhanced chemiluminescence blotting technique (ECL plus-Amersham Corporation, Piscataway, NJ, USA; RPN2133). Anti-PrP monoclonal antibody (mAb) 4H11, anti-PrP monoclonal antibody EP1802Y (Abcam, Waltham, MA, USA; ab52604), and anti-PrP monoclonal antibody 9A2 (Wageningen Bioveterinary Research, Lelystad, The Netherlands) were used to detect PrP signal either in brain homogenate or cell lysate. Anti-β-actin mAb (Sigma, Burbank, CA, USA; A5441), Peroxidase-conjugated immunoglobulins for immunoblot analysis (Jackson Immuno-research Lab, Inc., West Grove, PA, USA (goat anti-mouse HRP, 115-035-003)).

### 2.2. Cell Culture

CAD5 cells are a kind gift of Dr. S. Mahal (Scipps Institute, Jupiter, FL, USA) and were cultured in OptiMEM Glutamax medium (Thermo Fisher, Waltham, MA, USA) containing 10% bovine growth serum (Hyclone, Logan, UT, USA; SH30541.03) and penicillin/streptomycin in a 5% CO_2_ atmosphere. Cells were passaged every 5 days at a 1:10 dilution.

### 2.3. Brain Homogenate Samples

Camel prion-infected brain homogenate sample (241/576) was obtained from a prion-infected domestic camel (*C. dromedarius*). Mouse-adapted 22L scrapie strain brain homogenate was obtained from a terminally sick C57Bl/6 mouse. Bank vole-adapted 22L scrapie strain brain homogenate was obtained from a terminally sick bank vole. Mouse 22L and bank vole 22L brains were homogenized in PBS phosphate-buffered saline pH 7.4 (Life Technologies, Gibco, Carlsbad, CA, USA; 10010-023) using the MP Biomedicals fast prep-24 homogenizer (MP Biomedicals, Eschwege, Germany) at a final concentration of 10% (*w*/*v*). Aliquots were stored at −80 °C until further use.

### 2.4. Proteinase K Digestion of Brain Homogenate

Twenty µl of 10% brain homogenate was mixed with 20 µL 2X cell lysis buffer (final BH concentration is 5%). The mixture was divided into −PK and +PK tubes, 20 µL each. Two µL of 50X Pefabloc have been added to −PK tubes and PK to +PK tubes (2 µL of 1 mg/mL PK stock) and tubes were kept at 37 °C, 1 h, 450 rpm. 2 µL of 50X Pefabloc have been added to +PK tubes after the 1 h PK digestion followed by adding 20 µL of 3X SEB dye to −PK and +PK tubes. Tubes were boiled at 95 °C, 10 min, 1000 rpm. 5 µL for −PK and 10 µL for +PK were loaded onto the SDS-PAGE.

### 2.5. Primary Prion Infection

At day 1, 2 × 10^5^ cells were seeded in a 6-well culture dish. After 24 h. At day 2, the culture medium was removed, and cells were overlaid with 1% brain homogenate in an appropriate serum-free culture medium (450 µL). The mouse-adapted scrapie strain 22L was used to infect either WT-CAD5 or PrP-KO CAD5 cells. CAD5-camel-PrP was infected with the CPrD-brain homogenate sample (241/576). CAD5 cells expressing bank vole PrP were either infected with 22L-adapted bank vole brain homogenate or CPrD-brain homogenate sample (241/576). After 5 h incubation, 500 µL complete culture medium was added. The medium was removed 24 h later, and cells were washed once with PBS before fresh culture medium was added to the cells. For the detection of PrP^Sc^ upon primary prion infection, cells were lysed, and an aliquot of the cell lysate was subjected to PK-digestion and immunoblot analysis at each passage. The infection study was repeated with three independent replicates.

### 2.6. Ethics Statement

Mice and bank voles were kept in a controlled environment of humidity and temperature with an alternating 12 h light/dark cycle, with free access to food and drinking water until the time of euthanasia. The camel brain material came from an animal slaughtered for food consumption during routine sanitary inspection, and no experimental procedures were performed on animals for this study.

### 2.7. Cell Lysis, Proteinase K (PK) Treatment and Immunoblot Analysis

Immunoblot analysis was performed as previously described [22,23]. Confluent cells were lysed in cold lysis buffer (10 mM Tris-HCl, pH 7.5; 100 mM NaCl; 10 mM EDTA; 0.5% Triton X-100; 0.5% sodium deoxycholate (DOC)) for 10 min. Aliquots of post-nuclear and cleared lysates were incubated with PK (20 µg/mL) for 30 min at 37 °C. Digestion was stopped by the addition of proteinase inhibitors (0.5 mM Pefabloc) and directly precipitated with methanol. Samples without PK treatment were directly supplemented with proteinase inhibitors and precipitated with methanol. Precipitated proteins were re-dissolved in TNE buffer (50 mM Tris-HCl, pH 7.5; 150 mM NaCl; 5 mM EDTA). Aliquots were analyzed on 12.5% SDS-PAGE, electroblotted on PVDF membranes, and analyzed by immunoblot, using the ECL plus system.

### 2.8. Real-Time Quaking-Induced Conversion (RT-QuIC)

#### 2.8.1. Preparation of Recombinant PrP (rPrP) Substrate

The mature form of mouse PrP was cloned into pET41a expression vectors (EMD Biosciences, Gibbstown, NJ, USA) and expressed in *E. coli* Rosetta using the Express Autoinduction System (Thermo Fisher Scientific, Waltham, MA, USA). Inclusion bodies were prepared using the Bug Buster reagent (MilliporeSigma, Burlington, MA, USA) and solubilized in lysis buffer [guanidineHCl 8 M (MilliporeSigma)], sodium phosphate 100 mM, Tris–HCl 10 mM, pH 8.0 (Sigma-Aldrich, St. Louis, MO, USA) for 50 min at 23 °C and then centrifuged at 16,000× *g* for 5 min at 23 °C. Binding, refolding, and elution using an AKTA Explorer system have been described previously [24]. Mouse recombinant PrP is considered a versatile substrate for detecting various PrP^Sc^ types.

#### 2.8.2. RT-QuIC Assay

RT-QuIC was performed as described previously [25,26]. Briefly, reactions were set up in assay buffer containing 20 mM sodium phosphate (pH 6.9; Sigma-Aldrich), 300 mM NaCl (Sigma-Aldrich), 1 mM EDTA (Sigma-Aldrich), 10 μM Thioflavin T (Sigma-Aldrich) and 0.1 mg/mL recombinant mouse substrate. Quadreplicate reactions were seeded each time with 2 μL tenfold serial dilutions of either brain homogenate or cell lysate samples (seeds) in RT-QuIC seed dilution buffer (0.05% (*w*/*v*) SDS in 1× PBS). The plate was sealed with Nunc Amplification Tape (Nalge Nunc International, Rochester, NY, USA) and placed in a BMG Labtech FLUOstar Omega fluorescence plate reader (BMG LABTECH, Ortenberg, Germany) that was pre-heated to 42 °C for a total of 50 h with cycles of 1 min double orbital shaking (700 rpm) and 1 min resting throughout the assay time. The Thioflavin T fluorescence signals of each well were read and documented every 15 min for 200 cycles, then the values of the relative fluorescence units (RFU) were plotted as the average of octuplicate reactions. The cut-off was calculated based on the average fluorescence values of the negative control + 5 × SD.

### 2.9. Immunofluorescence and Confocal Laser Microscopy

Cultured cells were rinsed twice with PBS and fixed with 500 µL of 4% paraformaldehyde (GeneAll, Seoul, Republic of Korea; SM-P01-100) in PBS for 30 min at room temperature, followed by three washes with PBS. Cells were permeabilized with 0.1% Triton X-100 in PBS for 10 min. Cells were incubated for 1 h with a blocking solution, 5% goat serum in PBS. Cells were incubated with the primary antibody 4H11 (1:100) in blocking solution for 1 h, followed by 3 washes with PBS. Cells were incubated with secondary antibody (Alexa Fluor^TM^ 555 goat anti-mouse) for 1 h followed by 3 washes with PBS. Cells were mounted using mounting medium with DAPI (PermaFluor™, Thermo Scientific, Waltham, MA, USA;TA-006-FM) and were dried overnight at 4 °C. Confocal laser microscopy was carried out using a LSM 700 laser scanning microscope (Zeiss, Oberkochen, Germany).

### 2.10. Lentiviral Transduction

To insert different PrP genes, pWPI plasmid (Addgene#12254) was modified to have BamHI-SpeI-MluI-XmaI cloning sites (pWPI-BSMX). The PrP amplicons were inserted into EcoRI/BamHI cloning site and are expressed under EF-1-alpha promoter along with eGFP. Co-expression of the inserted gene and eGFP is due to having the Internal Ribosome Entry Site (IRES) sequence, (which is a translational enhancer that can recruit ribosomes and make two proteins from a single bicistronic mRNA. GFP expression was used for FACS enrichment of the transduced cells (Supplementary Figure S1A). Camel or bank vole PrP encoding lentiviruses were used to transduct CAD5-KO cells (Supplementary Figure S1B,C).

Lentivirus production was carried out using 293FT cells cultured to 90% confluency on five 15 cm^2^ cell culture plates. The cells were co-transfected with 112.5 μg of pWPI-PrP, 73 μg of psPAX2, and 39.5 μg of pMD2.G (all gifts from Didier Trono, Addgene, Watertown, MA, USA; plasmid #12260) using the PEIpro transfection reagent from Polyplus (Illkirch-Graffenstaden, France). pWPI-PrP is a lentiviral vector plasmid used for PrP gene delivery. psPAX2 is a 2nd generation lentiviral packaging plasmid that provides the essential viral proteins needed for the assembly of lentiviral particles. pMD2.G is an envelope plasmid that facilitates infection across a wide range of cell types. 293FT cells are known for their high transfection efficiency and robust production of lentiviral particles [27]. The cell culture media, containing the lentivirus, were harvested 48 h post-transfection. It was first centrifuged at 500× *g* for 5 min to remove cell debris and then filtered through a 0.45 μm filter. To concentrate the lentivirus, the filtered media was underlaid with a 10% sucrose solution in 1X PBS and subjected to ultracentrifugation at 12,000× *g* for 4 h at 4 °C. This method aligns with the optimized protocol described by Jiang et al. [28], which demonstrated that sucrose gradient centrifugation can efficiently produce high-titer lentivirus. The pellet was then resuspended in cold 1X PBS and aliquoted into 20 μL portions before being stored at −80 °C.

The physical titer of the lentivirus was measured using a qPCR Lentivirus Titer Kit (Applied Biological Materials Inc., Richmond, BC, Canada; No. LV900), according to the manufacturer’s instructions. This method estimates the total number of viral RNA particles, including both infectious (functional) and non-infectious particles. Consequently, may lead to an overestimation of the true number of infectious viral units [29]. To provide a more accurate measure of transduction efficiency, both the physical titer (determined by qPCR) and biological markers (eGFP expression) were used to calculate the multiplicity of infection (MOI), representing the ratio of infectious viral particles to target cells.

To verify viral functionality, HEK293 cells—a human embryonic kidney cell line widely used in viral research due to their high transduction efficiency and robust expression of transgenes—were grown to 70% confluency in 12-well plates in the presence of 8 μg/mL polybrene. Serial dilutions of the lentivirus were prepared and used to transduce these cells. eGFP expression, serving as a marker of successful transduction, was assessed 72 h post-transduction using an EVOS epifluorescence microscope (Thermo Fisher) [30]. This initial assessment confirmed efficient transduction and target gene expression.

To optimize lentiviral titer for PrP gene expression, CAD5 PrP-KO cells were plated in 12-well dishes at 5 × 10^4^ cells/well in 1 mL of complete media (Optimum-glutamax, 10% BGS, 1% antibiotics). After overnight incubation at 37 °C, the media was replaced with fresh media containing polybrene (4 μg/mL), and the cells (~30% confluent) were transduced with 1 µL, 3 µL, or 5 µL of lentiviral particles (pWPI-Camel^PrP^ or pWPI-BV^PrP^). The plate was centrifuged at 800× *g* for 10 min at room temperature (2100 rpm, rotor radius 14.7 cm), then incubated overnight at 37 °C. The following day, the virus-containing media was removed, and 1 mL of fresh media was added per well. The cells were then cultured for further expansion.

### 2.11. Fluorescence Imaging

Fluorescence imaging for HEK cells was initially carried out by the University of Calgary core facility to evaluate the efficiency of the produced lentiviral particles. For this purpose, HEK293T cells, grown to ~70% confluency (~300,000 cells per well), were transduced, and eGFP expression was assessed after 72 h. CAD5 PrP-KO cells were similarly transduced with 1 µL, 3 µL, and 5 µL of lentiviral particles in our lab, and fluorescence imaging was performed after three days to evaluate transduction efficiency (eGFP expression) and cell viability. The corresponding MOIs for each viral construct and volume are explained in Table 1.

### 2.12. Fluorescence-Activated Cell Sorting (FACS)

FACS analysis was done for the transduced PrP gene, which is co-expressed along with eGFP to select cells with the highest GFP expression. Cells were transferred to the Flow Cytometry Core Facility at the Cumming School of Medicine, University of Calgary. For sorting, the top 10% of GFP-positive single cells were selected. BDFACS-Fusion cell sorter to enrich GFP-positive cells. A detailed gating strategy was employed to ensure high purity. Mononuclear cells were first gated (P1) based on forward scatter (FSC) and side scatter (SSC) parameters to exclude debris. To eliminate doublets and aggregates, we further gated single cells using SSC-width (SSC-W) vs. SSC-height (SSC-H) and FSC-width (FSC-W) vs. FSC-height (FSC-H) plots. From the gated single-cell population, GFP-positive cells were identified (P2) and sorted into a collection tube.

### 2.13. DNA Extraction, Polymerase Chain Reaction (PCR) and Sequencing

DNA was extracted using the Qiagen DNeasy Blood & Tissue Kit (Qiagen, Hilden, Germany: cat # 69504) with 20 μL of 10% BH. The extracted DNA was screened for prion seeding activity via RT-QuIC. Upon confirming a negative result, the sample was used for PCR amplification. The *Prnp* open reading frame in exon 3 was amplified using a previously established protocol from [31]. PCR conditions were as follows: 5.5 μL of genomic DNA (equivalent to 100 ng of genomic DNA), 2 μL of 10 μM forward primer (5′–GCTGACACCCTCTTTATTTTGCAG–3′), 2 μL of 10 μM reverse primer (5′–GATTAAGAAGATAATGAAAACAGGAAG–3′), 10 μL of 10x Pfu buffer (Agilent, Santa Clara, CA, USA), 1 μL of 10 μM dNTP (Invitrogen, Carlsbad, CA, USA), 0.5 μL of Pfu polymerase (Agilent) and nuclease-free water in a total reaction volume of 50 μL. Conditions for the PCR were 4 min of initial denaturation at 94 °C, followed by 39 cycles of denaturation at 94 °C for 30 s, annealing at 63 °C for 30 s, and extension at 72 °C for 1 min, with a final extension at 72 °C for 10 min. No-template controls were included in every run to ensure no cross contamination of the sample. Amplified samples were run on a 2% agarose gel at 100 V for 1 h. Bands were visualized under UV light, excised using a clean blade, and amplified DNA was retrieved and purified using QIAquick Gel Extraction Kit (Qiagen, cat # 28704). Sequencing of products were performed at Eton Bioscience (San Diego, CA, USA) with the primers used in the PCR above. Data was analyzed using Geneious software version 2025.2.

### 2.14. Karyotyping and Chromosome Count

Karyotyping was performed on CAD5-wild-type and CAD5 knockout cell lines to assess their chromosomal stability and integrity and identify aneuploidy. Cells were submitted to the Centre for Genome Engineering, ES Cell Facility of the University of Calgary. Cells were incubated with 0.01 µg/mL of Colcemid^TM^ Solution (Gibco, Grand Island, NY, USA) for 30 min at 37 °C with 5% CO_2_ to optimize the visualization of well-condensed chromosomes by arresting cells in metaphase through preventing spindle formation during this stage. Then, the cells were trypsinized into single cells, resuspended and treated with 0.56% KCl for 6 min at room temperature. The hypotonic solution causes cells to swell, spreading the chromosomes for better resolution. Subsequently, they fixed with 3:1 Methanol: glacial acetic acid and dropped onto clean slides. Then the slides were air-dried on a warmer at 50 °C and stained with Giemsa dye, for better resolution. Finally, slides were examined under a microscope, and 40 intact spreads were counted for each cell line.

### 2.15. Statistical Analysis

Statistical analysis was performed using GraphPad Prism (Prism 7.05), employing either the unpaired two-tailed *t*-test for pairwise comparisons or one-way analysis of variance with the Tukey post hoc test for multiple comparisons. Statistical significance was expressed as follows: *, *p* ≤ 0.05; **, *p* ≤ 0.01; ***, *p* ≤ 0.001; ****, *p* ≤ 0.0001. The graphs were plotted using GraphPad Prism.

## 3. Results

### 3.1. Lentiviral Transduction of CAD5 KO Cells to Establish Camel and Bank Vole PrP Expressing Cells

We used a subclone of the gene-edited and mouse PrP-deleted CAD5 cells we have described previously [21]. Subcloning was done to exclude any remaining intact endogenous mouse PrP alleles. This is important as CAD5 cells are aneuploid, as verified by us by karyotyping analysis (Supplementary Figure S2).

**Figure 1:**
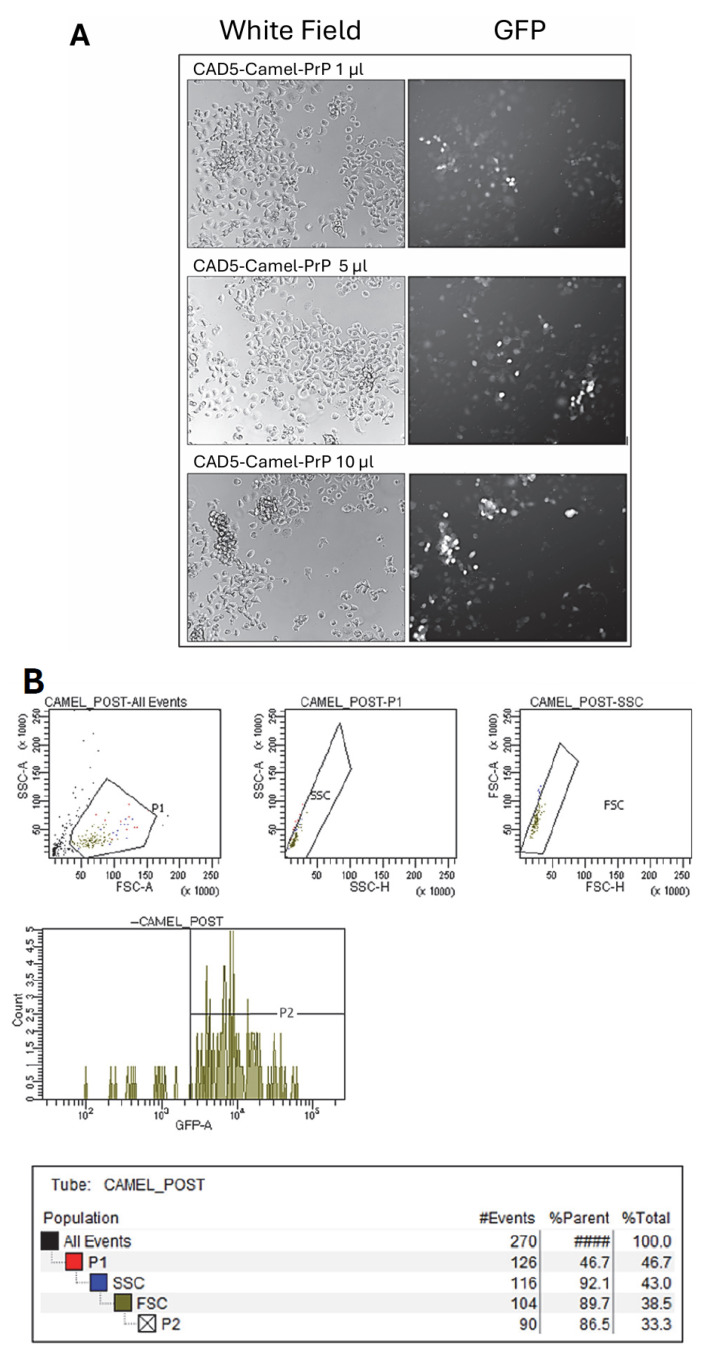
Fluorescence imaging and FACS analysis of CAD5-Camel-PrP transduced cells upon lentiviral transduction. (**A**) Fluorescence imaging of CAD5-Camel-PrP Cells. CAD5-PrP KO cells were transduced with 1 µL (**top panel**), 3 µL (**middle panel**), and 5 µL (**bottom panel**) of lentiviral particles encoding camel-PrP. The left panels show bright-field images, while the right panels display the corresponding fluorescence images. Fluorescence intensity correlates with the volume of lentiviral particles used, reflecting the level of PrP expression. The 1 µL condition shows minimal and sparse GFP fluorescence, indicating low transduction efficiency. The 3 µL condition yields increased GFP expression with improved distribution across the cell population. The 5 µL condition demonstrated stronger fluorescence expressed as a more uniform GFP signal. (**B**) CAD5 cells expressing camel-PrP upon lentiviral transduction. The top panels show scatter plots for all events, P1 gating, and SSC (side scatter) versus FSC (forward scatter), and histograms of GFP fluorescence intensity, with the P2 gate indicating cells with high GFP expression (**middle panel**). The tables summarize the number of events and GFP statistics for each population.

CAD5 knocked-out cells were transduced with either camel or bank vole PrP-expressing lentivirus that also express eGFP. To assess the functionality of the viral particles and MOI to be used, HEK293 cells were initially transduced with different titers of the synthesized lentivirus. HEK293 cells were transduced with 1 µL, 3 µL, and 10 µL of either bank vole-PrP or camel-PrP encoding lentivirus. The corresponding MOIs for each viral construct and volume are explained in Table 2. We assessed eGFP expression in fluorescence analysis (Supplementary Figure S3). Next, CAD5 PrP-KO cells were transduced with 1 µL, 3 µL, and 5 µL of either bank vole-PrP or camel-PrP expressing lentivirus. The corresponding MOIs for each viral construct and volume are explained in Table 1. Immunofluorescence analysis was performed for both types of cells to ensure eGFP expression (Figure 1A, Supplementary Figure S4A). Fluorescent-activated cell sorting for eGFP was used to enrich for CAD5 cells with high camel-PrP and bank vole-PrP expression, consequently (Figure 1B, Supplementary Figure S4B). Following the first round, a second round of FACS was conducted to further enrich for the cells with high eGFP expression. Our data show enrichment of eGFP expression following FACS analysis, when comparing the histograms of pre (P1) and post (P2) populations. In camel-PrP expressing cells, P1 was 46.7% while P2 was 86.5% (Figure 1B). For bank vole-PrP expressing cells, P1 was 51.6% while P2 was 90.0% (Supplementary Figure S4B). Taken together, these data demonstrate a homogeneous and highly enriched cell population. High eGFP expression indicates successful transduction and PrP expression in these cells, and the P2 population with the highest eGFP expression was selected for further experiments.

**Figure 2:**
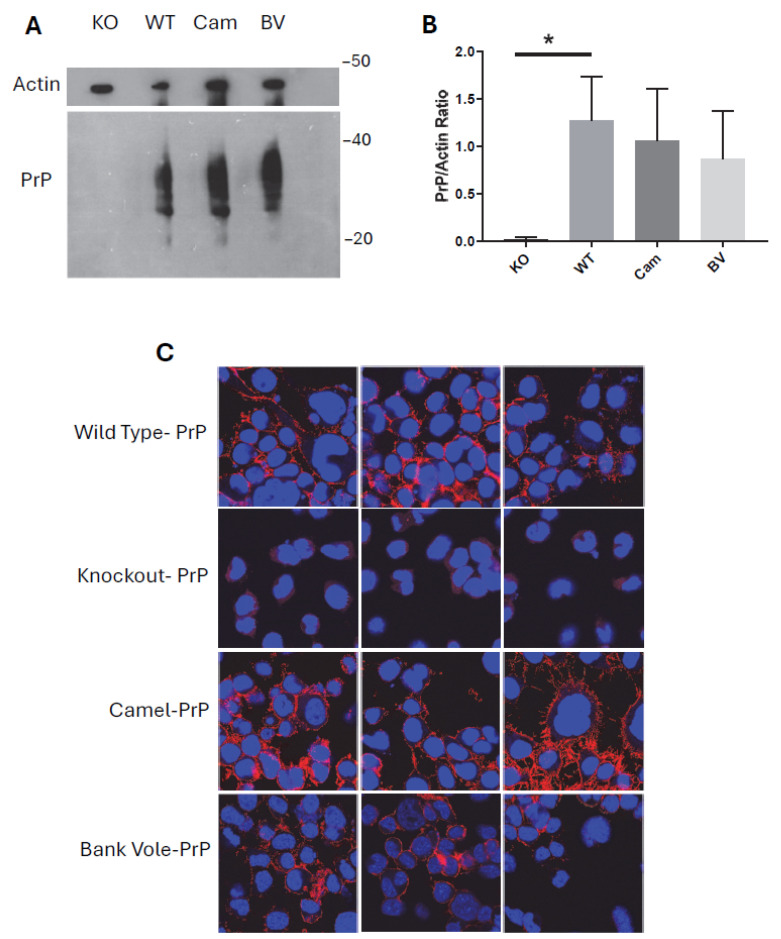
Characterization of CAD5 transduced cells. (**A**) PrP immunoblot for wild-type (WT), knockout (KO), CAD5 cells expressing camel-PrP (Cam) or CAD5 cells expressing bank vole-PrP (BV). 4H11 monoclonal antibody was used to detect PrP. Actin was used as a loading control. (**B**) Densitometric analysis representing the PrP^C^ to actin ratio as shown in the immunoblot of panel (**A**). * indicates *p* ≤ 0.05. (**C**) PrP immunofluorescence for wild-type, knockout, CAD5 cells expressing camel-PrP or CAD5 cells expressing bank vole-PrP. Cells were stained with the monoclonal anti-PrP antibody 4H11 (red), and nuclei were stained with DAPI (blue).

For confirmation of PrP expression in the transduced CAD5 cells, we performed Western blot analysis on post-nuclear lysates. CAD5-KO cells did not show any PrP positive signal, confirming that these cells do not express PrP. CAD5-WT cells were used as a positive PrP control. CAD5 cells reconstituted with bank vole-PrP and camel-PrP showed typical bands for PrP, indicating successful reconstitution of PrP expression in these cells after the transduction process (Figure 2A). Densitometric analysis did not demonstrate any significant difference between the PrP expression level in the wild-type cells compared to the transduced cells (Figure 2B). Next, we performed immunofluorescence analysis using a PrP-specific antibody to confirm the immunoblotting results. This revealed a strong and specific PrP signal, mostly at the plasma membrane, in both CAD5-bank vole-PrP and CAD5-camel-PrP transduced cells. The signal was comparable to the PrP signal in wild-type cells. (Figure 2C).

### 3.2. Prion Infection of Transduced CAD5 Cells

**Figure 3:**
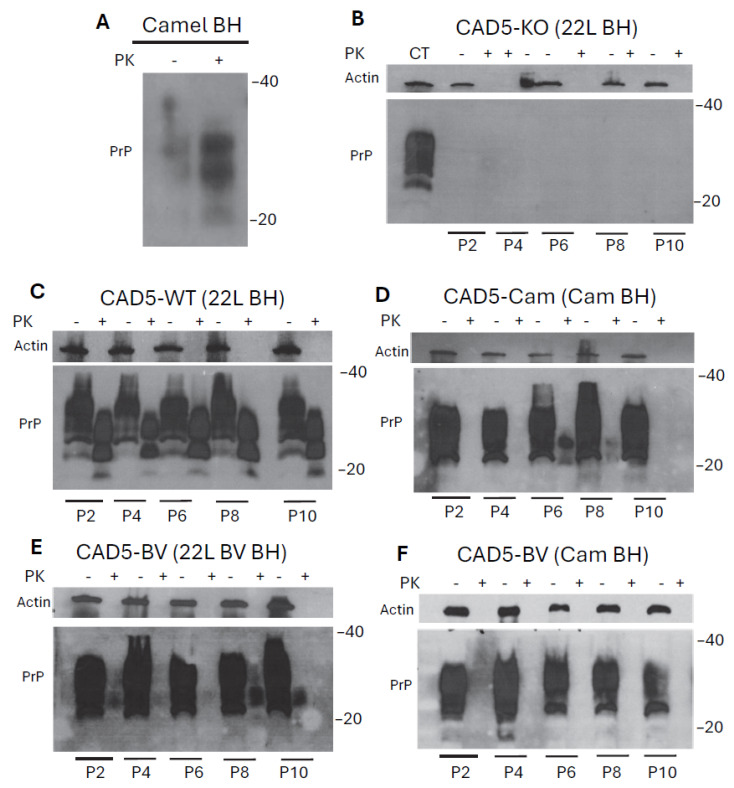
Immunoblot of CAD5 cells after prion infection challenge. (**A**) Immuno-blotting of CPrD-positive brain homogenate sample before and after PK digestion. The anti-PrP mAb 9A2 was used. (**B**–**F**) Immunoblot from CAD5 cell lysates. Cells are PrP-KO CAD5 cells infected with 22L mouse prions (negative control) (**B**), CAD5 PrP wild-type cells infected with 22L mouse prions (positive control) (**C**), CAD5 cells expressing camel-PrP infected with CPrD brain homogenate (**D**), CAD5 cells expressing bank vole-PrP infected with bank vole brain homogenate (**E**), or CAD5 cells expressing bank vole-PrP infected with CPrD camel brain homogenate (**F**). Cells in B and C were infected with 22L brain homogenate of a terminally sick mouse. Cells in D and F were infected with CPrD brain homogenate. Cells in E were infected with 22L-infected bank vole brain homogenate from a terminally sick bank vole. Western blotting was done after passages 2, 4, 6, 8, and 10 of infection, and samples were treated with or without PK digestion. mAb 4H11 was used to detect PrP. Actin was used as a loading control. Data shown is representative for three independent experiments.

The CPrD-positive brain homogenate was from an infected dromedary camel (*Camelus dromedarius*) from Algeria [15]. We first confirmed the presence of PrP^Sc^ in the provided sample after proteinase-K digestion (Figure 3A). Upon characterization of the CPrD-positive brain homogenate using anti-PrP mAb 9A2, we investigated whether camel PrP–expressing CAD5 cells can propagate camel prions. We used different types of CAD5 cells (i.e., wild-type, PrP-knockout, CAD5-camel-PrP or CAD5-bank vole-PrP expressing cells) and challenged them with different types of prion-infected brain homogenates. Wild-type CAD5 was infected with 22L brain homogenate and used as a positive control. CAD5 PrP-Knockout cells were infected with 22L brain homogenate and used as a negative control. CAD5-camel-PrP cells were infected with camel brain homogenate. Finally, CAD-bank vole-PrP cells were infected with 22L bank vole prions to prove that cells can propagate prions if infected with bank vole prions or were infected with camel brain homogenate to test the ability of bank vole-expressing cells to accept prion infection from a different species. Of note, all brain homogenates used in this study were tested by RT-QuIC to ensure prion conversion activity (Supplementary Figure S5A). After infection, the cells were passaged several times, with one passage corresponding roughly 5 days, and cell lysates were prepared after every other passage.

Cell lysates were subjected to PK digestion (20 µg/mL for 30 min) and analyzed by immunoblot using anti-PrP mAb 4H11, which exhibits robust cross reactivity and has been able to detect different types of PrP, including mouse, bank vole and camel PrP. Our results showed that CAD5 cells expressing camel-PrP infected with camel brain homogenate showed PrP^Sc^ signals after PK digestion at passages 6 and 8 post-infection (Figure 3D, Supplementary Figure S5B). CAD5 cells expressing bank vole-PrP infected with camel brain homogenate did not show any PrP^Sc^ signal over the different passages of the experiment (Figure 3F). Note that bank vole-PrP expressing cells were able to propagate prions when infected with the matched tissue, 22L-infected bank vole brain homogenate, and this signal persisted until the end of the experiment (Figure 3E). Our positive control wild-type CAD5 cells exhibited a sustained, strong PrP^Sc^ signal that was maintained for the duration of the experiment, indicating a persistent infection (Figure 3C). Our negative control, PrP Knockout CAD5, did not show any PrP^Sc^ signal throughout all the passages (Figure 3B). Notably, mAb 4H11 could be used in the infection study for all the transduced cells, as it successfully detects the signal of mouse, camel and bank vole PrP in CAD5 cells.

**Figure 4:**
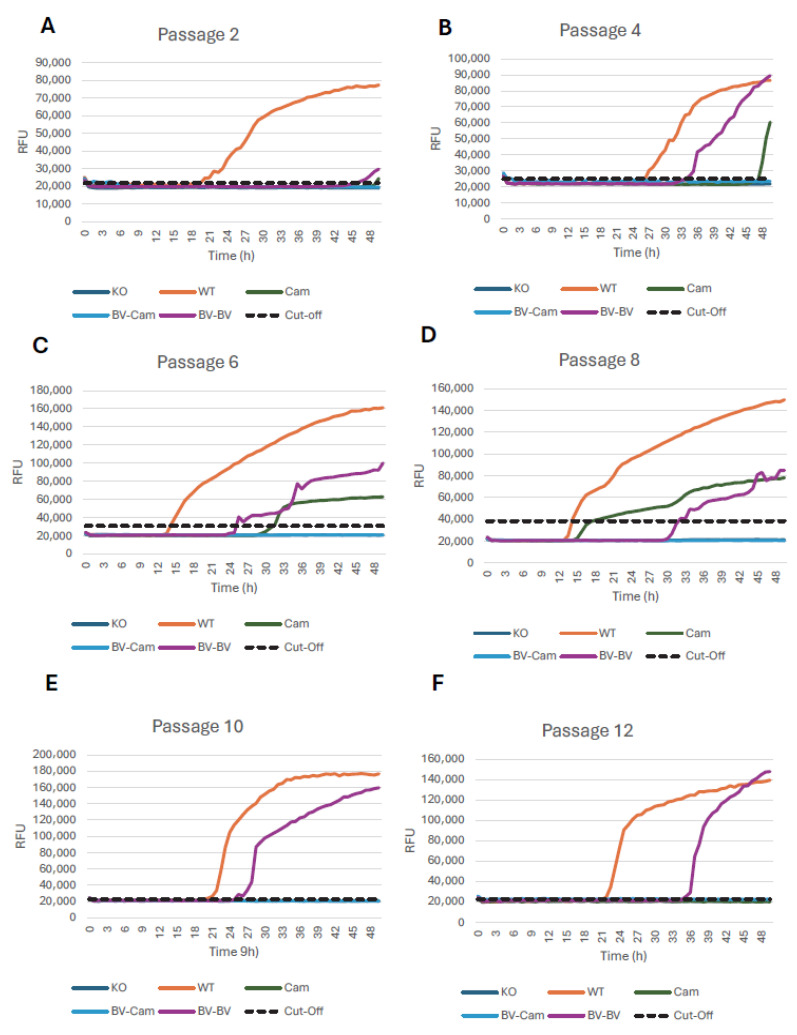
RT-QuIC of CAD5 cells after prion infection. (**A**–**F**) RT-QuIC from CAD5 cell lysates. CAD5-KO (dark blue colour) and CAD5-WT (orange colour) cells were infected with 22L brain homogenate of a terminally sick mouse. CAD5 cells expressing camel-PrP were infected with CPrD-positive brain homogenate (light blue colour). CAD5 cells expressing bank vole-PrP were infected with either 22L bank vole-adapted brain homogenate from a terminally sick bank vole (violet colour) or CPrD-positive brain homogenate (green colour). RT-QuIC was done after passages 2, 4, 6, 8, 10, and 12 post-infection. Mouse recombinant PrP was used as a substrate. The average increase in thioflavin-T fluorescence of replicate wells is plotted as a function of time. The *y*-axis represents RFU, and the *x*-axis represents time (h). Assay was performed in quadruplicates, with >2 replicates above the cut-off considered positive. The data represent four biological replicates.

Next, the prion conversion activity of cells was tested using the RT-QuIC technique. Mouse recombinant PrP was used as RT-QuIC substrate, as it is in our hands one of the best-performing substrates for RT-QuIC. It converts efficiently into amyloid in the presence of seeds and produces strong and reliable Thioflavin-T fluorescence signals, working reliably across many species and prion strains. Overall, immunoblot results matched our RT-QuIC conversion activity data. RT-QuIC data showed that CAD5 cells expressing camel-PrP infected with CPrD-positive homogenate were positive at passages 4, 6 and 8, losing conversion activity in passage 10 (corresponding to ~50 days post infection) and showing a very weak conversion activity in passage 2. CAD5-bank vole-PrP cells infected with camel brain homogenate did not show prion conversion activity over the analyzed passages. CAD5-bank vole-PrP expressing cells consistently showed prion conversion activity when infected with the matched bank vole tissue. The positive control, 22L infected wild-type CAD5 cells, demonstrated conversion activity that was maintained for the duration of the experiment, indicative of persistent infection. The negative control, PrP knockout CAD5 cells (22L infected), did not show any conversion activity throughout all the passages (Figure 4A–F). Quantitative analysis was done for established RT-QuIC criteria like maximum fluorescence, area under the curve and time to threshold, to demonstrate the changes in seeding activity across the passages analyzed (Supplementary Figures S6–S8). For example, time to threshold considered a reliable marker for positivity in RT-QuIC analysis was consistently negative for non-reconstituted PrP KO cells and bank vole-PrP expressing cells inoculated with camel prions, and positive for wild-type cells infected with 22L prions from passage 2 to 12. For cells expressing camel-PrP and inoculated with camel prions, signals increased from early to middle passages and were not detectable at late passages. Unfortunately, we could not test for specific prion infectivity of cultured cells in animal bioassays.

Taken together, these results show that gene-edited CAD5 cells can propagate camel prion propagation when expressing the camel prion protein.

## 4. Discussion

### 4.1. Camel Prion Disease

Camel prion disease (CPrD) is the latest member of animal prion diseases and is a fatal and likely infectious neurodegenerative disorder found in dromedary camels. As typical for prion diseases, CPrD affects the camel’s nervous system, leading to progressive behavioural changes and movement difficulty [15,16]. Although research is still limited, CPrD is important because camels are widely used for meat and milk in some regions, raising concerns about animal health, food safety, and the need for monitoring and further study of CPrD prion transmission.

Camel prion disease may result from one of two possibilities: it could be acquired through prion-contaminated feed, though this is unlikely since these camels are not commonly raised on such feeds, and no evidence of BSE or scrapie has been reported in the affected regions, such as Algeria and Ethiopia. The other possibility is an initially sporadic origin, caused by the spontaneous misfolding of the prion protein. Supporting this, the PrP^Sc^ signature in CPrD differs from that of BSE or scrapie, pointing to a non-related mechanism. Considering that genetic studies found no differences or suggestive PrP polymorphisms affecting susceptibility in the *prnp* gene among dromedary camel populations across Algeria and Ethiopia, the question of susceptibility to lateral infection is open. This genetic uniformity suggests that dromedaries may lack evolutionary adaptations to resist prion diseases, highlighting potential risks for this critical livestock species in Africa, Asia, and the Middle East [15,17]. Of note, the CPrD isolate used here was also encoding a wild-type dromedary camel *prnp* sequence.

### 4.2. Studying Prions in Cell Culture Models, Including Gene-Edited Cells

Using established cell lines allows prion researchers to study prion infection in a controlled, safe, and reproducible laboratory environment. Cell lines can be infected with prions and observed over time, making it easier to study the molecular and cellular mechanisms of how prions convert PrP^C^ into PrP^Sc^ and spread within and between cells. Importantly, only some cell lines, of neuronal or non-neuronal origin, can be infected persistently and only with certain types of prions [32]. In other words, cellular prion infection depends on the species of prions and subtype/strain. For example, we do not have cell lines that robustly propagate human or bovine prions. In addition, cell line models are useful for testing potential anti-prion treatments, screening drugs, and comparing how different prion strains behave at the cellular level [33,34,35,36]. Interestingly, all persistently prion-infected models are transformed and/or immortalized cell lines that have acquired structural and numerical chromosome abnormalities over time, a fact that does apparently not compromise their ability to propagate prions. Nevertheless, it is recommended to use lower passage numbers, if known and possible, for new infection studies, as we have done here. Of note, aneuploidy is important in CRISPR/Cas-9 based knockout experiments and it is important to verify that every allele is accordingly edited not to have a wild-type PrP allele in the background.

PrP knockout cells, either derived from knockout mice or generated by gene-editing, do not express the normal prion protein that is required for prion infection and replication. Starting with cells that lack PrP provides a “clean background,” so any prion-related effects can be clearly linked to the PrP that is later added. By stably transducing these knockout cells with other PrPs—such as PrPs from different species or PrPs with specific mutations or polymorphisms—we can study how changes in PrP affect susceptibility to prion infection, prion conversion, and prion strain disease mechanisms. This approach allows studying exogenous prions without interference from the endogenous PrP, which would negatively interfere with prion infection [37,38]. For example, a mouse cell line expressing endogenous murine PrP can only be infected with mouse-adapted scrapie prions, and not with cervid, camel or human prions. A mouse cell line where the endogenous mouse PrP is deleted and replaced with a cervid PrP can be infected with cervid prions [20,21]. Cells expressing a bank vole PrP can be an exception to this rule, as bank vole PrP is considered a universal acceptor for prion replication [39,40]. In line with this, it has been reported that CAD5-PrP-knockout cells reconstituted with hamster PrP allowed the successful propagation of hamster prion strains [41]. In later work, these authors reconstituted CAD5-PrP knockout cells with mouse PrP, bank vole PrP, and hamster PrP [41,42]. Based on their results, CAD5 cells expressing bank vole PrP were susceptible to both mouse and hamster prions, while the cells expressing mouse PrP or hamster PrP could only be infected by species-matched prions. A similar strategy was used by us for generating cell models (CAD5 and MEF) for CWD prions, using bank vole as a universal acceptor [21]. However, none of these studies investigated camel prions, highlighting a gap in the available models for this newly emerging prion disease.

As done before [21], we used CAD5 knockout cells and generated CAD5 cells expressing only camel PrP. Cells were characterized for camel PrP expression using immunoblotting and immunofluorescence techniques. Here, we report for the first time prion infection in camel PrP-expressing CAD5 neuronal cells when challenged with CPrD-positive brain homogenate. This was confirmed by Western blotting for PrP^Sc^ and prion conversion activity with RT-QuIC at various passages post infection, both showing positive results for passages 6 and 8, representing 30 to 40 days post infection. This increase over time excludes residual PrP^Sc^ inoculum, as does the fact that both immunoblot and RT-QuIC were completely negative for the inoculated KO cells, even at passage 2. Not unexpected, infection in CPrD-challenged camel-PrP expressing CAD5 cells was maintained for a limited period only, and then apparently lost. One explanation for this limited propagation time is that newly generated CPrD prions did not adopt to a conformation that is resistant to cellular clearance mechanisms, resulting in shifting the delicate balance between cellular prion propagation and prion clearance to the latter over time [43,44]. This apparent limitation can be usually overcome by doing single-cell cloning to enrich for cells that facilitate stable, persistent prion infection, either before or during the infection experiment.

Prion transmission between species is inhibited by the “species barrier” phenomenon [45,46,47], which makes it challenging for prions to spread across different species and makes it difficult to study certain prions in cell culture models. Since prion diseases spread through templating, where PrP^Sc^ acts as a template for PrP^C^ misfolding, a species barrier occurs when there is a mismatch between recipient PrP and exogenous PrP^Sc^, preventing prion propagation [48,49]. It has been reported that bank voles (*Myodes glareolus*) do not exhibit the species barrier phenomenon because of their unique PrP sequence, which is highly prone to spontaneous and template-based misfolding [39,40]. As a result, bank voles could be considered a universal prion acceptor and would be susceptible to a variety of prion species. In line with this, it has been reported that animal models expressing bank vole PrP are susceptible to diverse prions [50]. As done conceptually before by us and others, we established a bank vole PrP expressing neuronal cell line using stable lentiviral transduction to test bank vole PrP expressing cells as a universal acceptor of camel prions. When challenged with brain homogenate from bank voles infected with 22L prions, mouse prions adapted to bank voles, cells were able to propagate these ‘bank vole’ prions. When we infected these cells with CPrD-positive brain homogenate, the cells were unable to propagate camel prions, at least in a way we should have detected in our immunoblot and RT-QuIC studies. Our data indicate that bank vole PrP is not an acceptor for CPrD prions coming from a camel brain homogenate in this specific cell model. Interestingly, when using bank vole brain as a substrate in PMCA to test prion amplification of the CPrD-positive camel brain homogenate, this was positive. Upon performing PMCA using mouse brain homogenate as a substrate, no amplification was observed after three rounds of PMCA. This discrepancy could be due to the fact that PMCA is a cell-free system, whereas CAD5 is a much more complex and living cellular system, propagating prions and also degrading them. Our data indicate that while bank vole PrP can support CPrD prion conversion under cell-free amplification conditions, it does not permit productive prion propagation in our gene-edited and re-constituted CAD5 cells, highlighting a cell model-specific barrier distinct from sequence compatibility alone.

Taken together, we generated a PrP-knockout in CAD5 neuronal cells, followed by lentiviral reconstitution with camel or bank vole PrP. Camel PrP-expressing CAD5 cells clearly facilitated the de novo propagation of camel prions, although productive infection was lost over time. We strongly believe that losing the PrP^Sc^ signal was due to the heterogeneous and not single cell-cloned nature of the cell population. We could not use antibiotic selection for PrP-expressing cells and relied on FACS-based enrichment only. In addition, only a subset of cells likely supported initial and continued prion replication. Over time, non-permissive cells can outgrow permissive ones and dilute out the infected subpopulation. Another factor could be response to cellular clearance mechanisms like autophagy and lysosomal machineries that might be different for camel prions and mouse and bank vole-derived prions, with the latter ones performing better in this specific experimental system.

To our best knowledge, this is the first cell line model that has been infected with camel prions, making it a valuable model for studying camel prion disease. However, establishing a persistently infected and robust cell model will require further refinement. Approaches such as single-cell cloning to isolate subclones with enhanced susceptibility to and/or maintenance of prion infection, or re-boosting infection by repeated prion exposure, could help generate a more stable prion-infected cell line. Given the recent emergence of camel prion disease, such models are crucial for understanding the underlying infection mechanisms, species barriers, and development of potential therapeutic strategies.

## Figures and Tables

**Table 1:** CAD5 PrP-KO cells were transduced with 1 µL, 3 µL, and 5 µL of either bank vole-PrP or camel-PrP lentivirus. The corresponding MOIs for each viral construct and volume are explained in the Table.

	Functional Titre (IU/mL)	MOI (5 µL)	MOI (3 µL)	MOI (1 µL)
pWPI-BV^PrP^	8.99 × 10^7^	0.5	1.4	2.5
pWPI-Cam^PrP^	1.47 × 10^8^	0.8	2.4	4.1

**Table 2:** HEK293 cells were transduced with 1 µL, 3 µL, and 10 µL of either bank vole-PrP or camel-PrP lentivirus. The corresponding MOIs for each viral construct and volume are explained in the Table.

	Functional Titre (IU/mL)	MOI (1 µL)	MOI (3 µL)	MOI (10 µL)
pWPI-BV^PrP^	8.99 × 10^7^	0.4	1.2	3.9
pWPI-Cam^PrP^	1.47 × 10^8^	1.0	3.1	10.2

## Data Availability

The datasets generated during and/or analyzed during the current study are available from the corresponding author on request.

## Supplementary Materials

The following supporting information can be downloaded at: https://www.mdpi.com/article/10.3390/pathogens15050472/s1, Figure S1: Lentiviral transduction vector and gene sequence for transduction; Figure S2: Karyotyping and chromosome count of CAD cells; Figure S3: Lentiviral titration in HEK293 cells; Figure S4: Fluorescence imaging and FACS analysis of CAD5-bank vole-PrP transduced cells upon lentiviral transduction; Figure S5: RT-QuIC analysis of brain homogenates used in the infection experiment; Figure S6: Maximum fluorescence intensity for RT-QuIC analysis after infection; Figure S7: The area under the curve for RT-QuIC analysis after infection; Figure S8: Time to threshold values for RT-QuIC analysis after infection.
