# Supplementary material for: A Neuronal Cell Line Model for Studying Camel Prions
Source: Pathogens. 2026 Apr 27;15(5):472. doi: 10.3390/pathogens15050472 (PMC13209568; doi:10.3390/pathogens15050472)
Supplement: Supplementary file 1 [file pathogens-15-00472-s001.zip › pathogens-4241663-supplementary.pdf]

Supplementary Figure 01

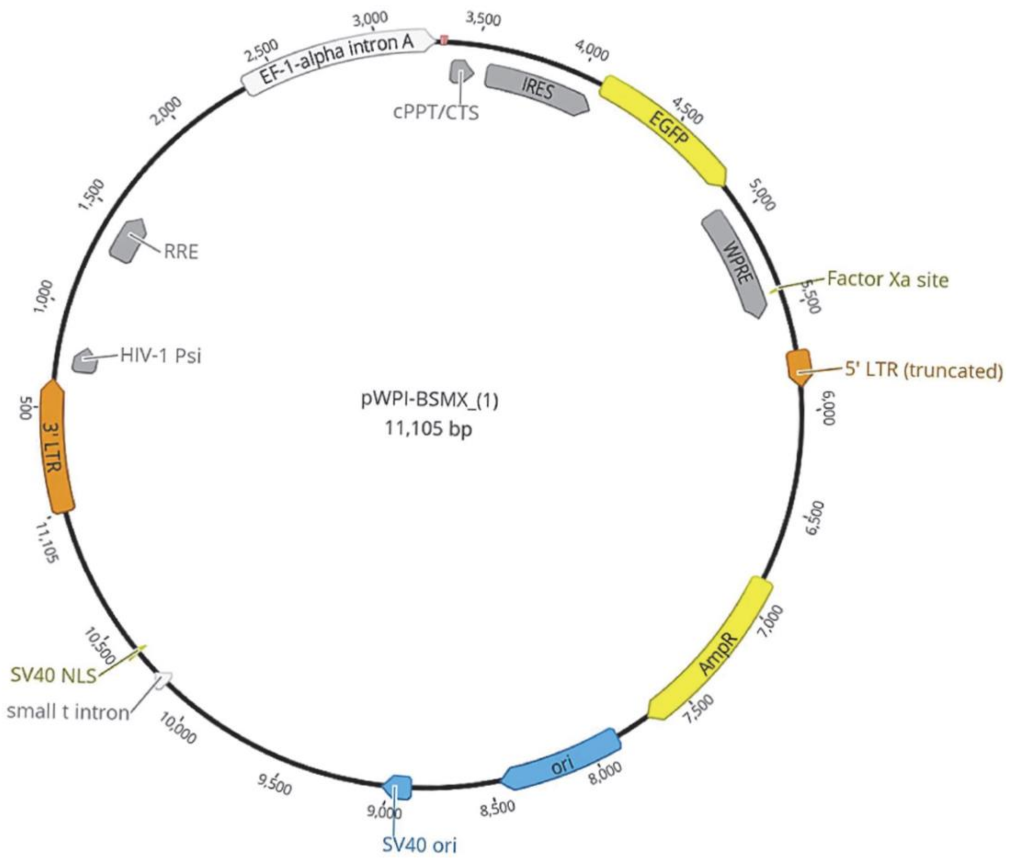

A

B **Camel_PrP_ORF** **768 bp**

```
  1 atggtgaaaa gccacatggg cagctggatc ctggttctct ttgtggtcac gtggagtgac
 61 gtgggcctgt gcaagaagcg cccaaagcct ggaggaggat ggaacactgg ggggagccga
121 tacccagggc agggcagtcc tggaggcaac cgctatccac cccagggagg gggcggctgg
181 ggtcagcccc acggaggagg ctggggtcag ccccacggag gcggctgggg tcaaccccac
241 ggaggcggct ggggccagcc ccatggtgga ggctggggtc aaggtggtgg cgcccacggt
301 cagtggaaca agcccagtaa gccgaaaacc agcatgaagc acgtggcagg agctgctgca
361 gctggggcag tggtaggggg ccttggtggc tacatgctgg ggagtgccat gagcaggccc
421 cttatacatt ttggcaacga ctatgaggac cgttactatc gagaaaacat gtaccgttac
481 cccaaccaag tgtactacaa gccagtggat cagtacagca accagaacag cttcgtgcat
541 gactgcgtca acatcacagt caaacagcac acggtcacca ccaccaccaa gggggagaac
601 ttcaccgaga ccgacgtcaa gatgatggag cgcgtagtgg agcaaatgtg catcacccag
661 taccagagag agtaccaggc ttcgtacggc agaggggcca gtgtgatctt ctcctcccct
721 cctgtgatcc tcctcatctc tttcctcatt ttcctcatag tgggttag
```

BankVole_PrP_ORF 757 bp

```
  1 atggcgaacc tcagctactg gctgctggca ttctttgtga ccacatggac tgatgtgggt
 61 ctctgcaaga agcggccaaa gcctggaggg tggaacactg gtggaagccg ataccctggg
121 cagggcagcc ctggaggcaa ccggtaccca cctcagggtg gtggtacctg ggacagccc
181 catggcggtg gctggggaca gcctcacggt ggtggttggg gtcagcctca cggtggcggt
241 tggggtcaac cccatggcgg cggctggggt caaggaggtg gcacccacaa tcagtggaac
301 aagcccagta agccaaaaac caacatgaag catgtggcag gcgctgccgc ggctggggca
361 gtggtggggg gcctgggtgg ctacatgctg gggagcgcca tgagcaggcc catgatccat
421 ttcggcaatg actgggagga ccgctactac cgtgaaaaca tgaaccggta ccctaaccaa
481 gtgtactacc ggccggtgga ccagtacaac aaccagaaca acttcgtgca cgattgcgtc
541 aacatcacca tcaagcagca tacagtcacc actaccacca aggggagaa cttcacggag
601 accgacgtca agatgatgga gcgcgtggtg gagcagatgt gcgtcaccca gtatcagaag
661 gagtcccagg cctactacga agggagaagt tcccgcgccg tgctgctctt ctcatcaccg
721 cctgtgatcc tcctcatttc cttcctcatc ttcctga
```

Supplementary Figure S1: Lentiviral transduction vector and gene sequence for transduction. (A) pWPI-BSMX Transduction Vector map. The pWPI-BSMX vector is a modified version of the pWPI plasmid designed to express PrP gene alleles under the control of the EF-1 alpha promoter along with eGFP. The vector includes an Internal Ribosome Entry Site (IRES) for bicistronic expression of the PrP gene and eGFP. **(B and C)** Open reading frame (ORF) of camel, and bank vole PrP sequence, which is inserted into the pWPI-BSMX vector.

Supplementary Figure 02

A

WT Spreads	%	No of Chr
1	1.85%	91
2	3.70%	109
6	11.11%	110
11	20.37%	112
2	3.70%	114
5	9.26%	116
1	1.85%	118
4	7.41%	120
2	3.70%	122
1	1.85%	124
1	1.85%	125
2	3.70%	126
1	1.85%	127
6	11.11%	130
1	1.85%	131
5	9.26%	132
1	1.85%	134
1	1.85%	135
1	1.85%	230
54		

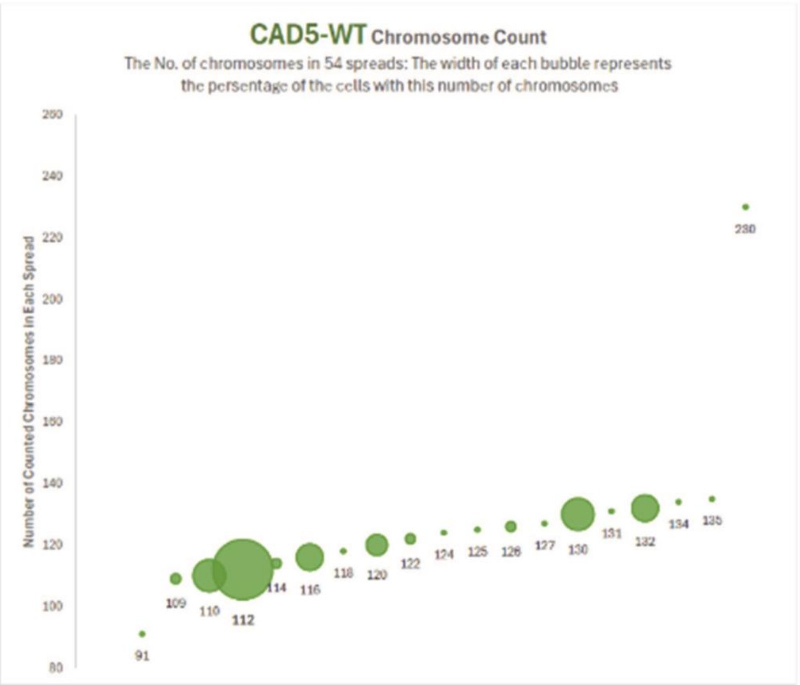

B

KO Spreads	%	No of Chr
2	4.17%	107
1	2.08%	109
3	6.25%	110
5	10.42%	111
6	12.50%	112
6	12.50%	114
3	6.25%	115
3	6.25%	116
2	4.17%	117
2	4.17%	118
3	6.25%	120
3	6.25%	126
1	2.08%	127
5	10.42%	130
1	2.08%	134
1	2.08%	234
1	2.08%	254
48		

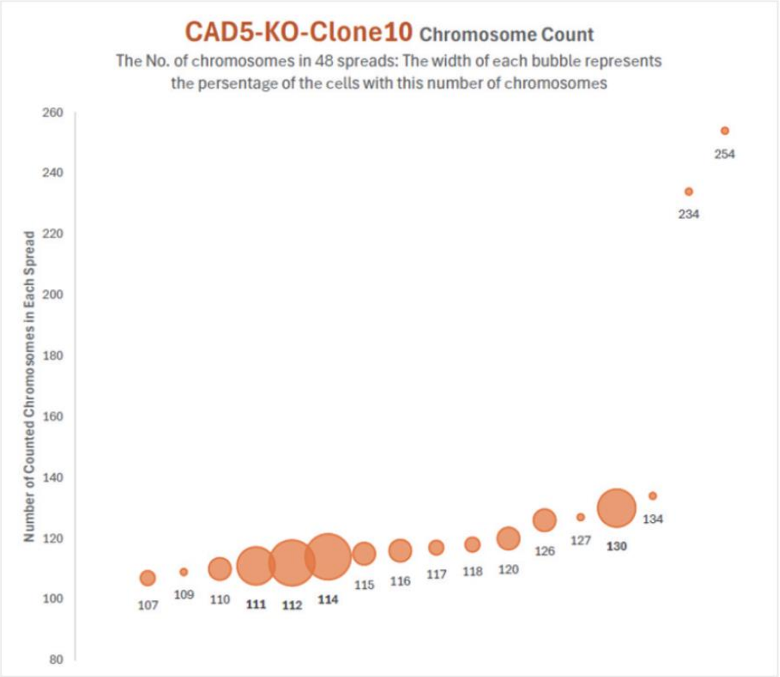

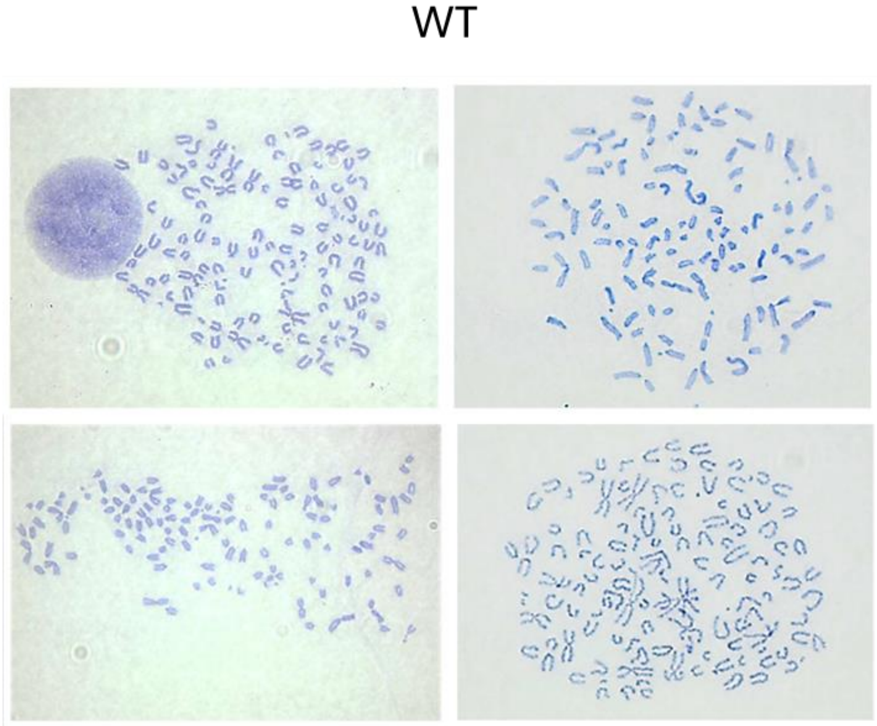

C

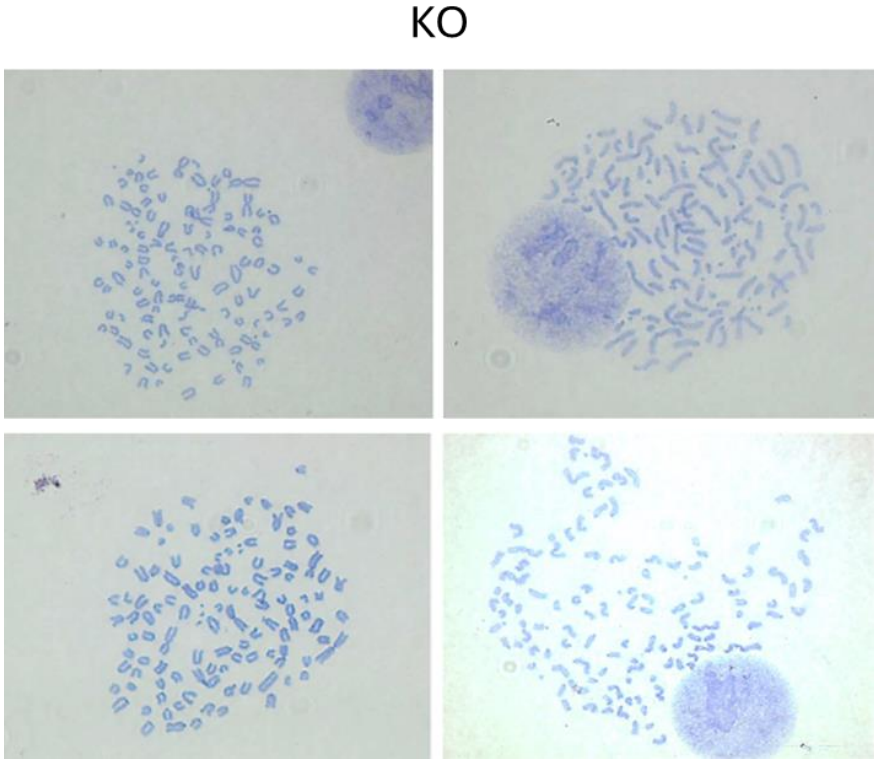

D

Supplementary Figure S2: Karyotyping & chromosome count of CAD cells. (A and B) Chromosomal count summary of CAD5 wild type **(A)** and CAD5-KO cells **(B)**. The summary is based on 54 spreads. The table shows the percentage of spreads for each chromosomal count, while the bubble chart on the right visualizes this data, with the width of each bubble representing the percentage of spreads with the corresponding number of chromosomes. The data reveal a wide range of chromosomal counts, indicating aneuploidy in the CAD5 cell line. The majority of spreads exhibit chromosomal counts between 110 and 130. **(C)** Representative metaphase chromosome spreads from karyotyping of CAD5 wild-type and **(D)** CAD5-KO cells.

Supplementary Figure 03

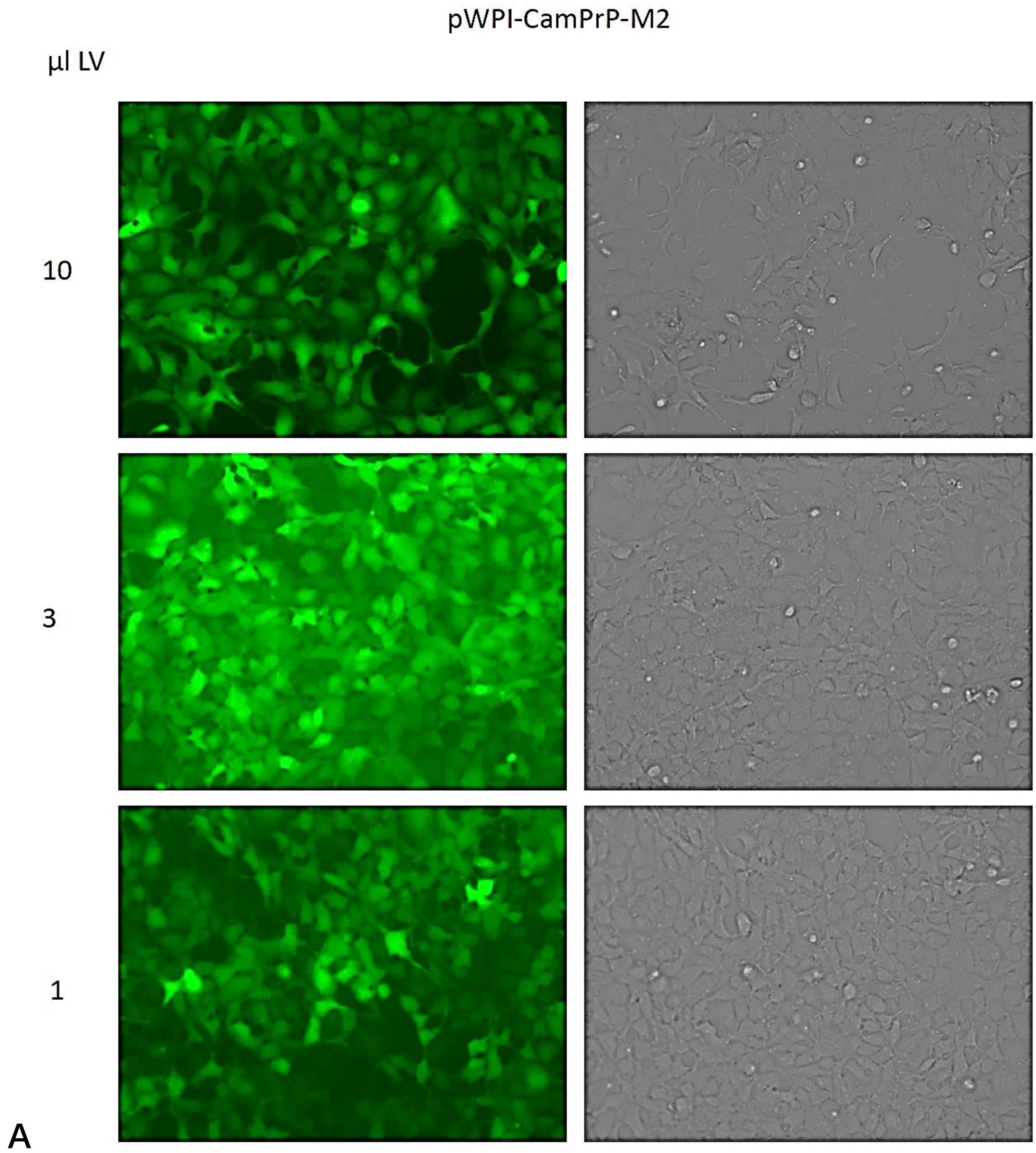

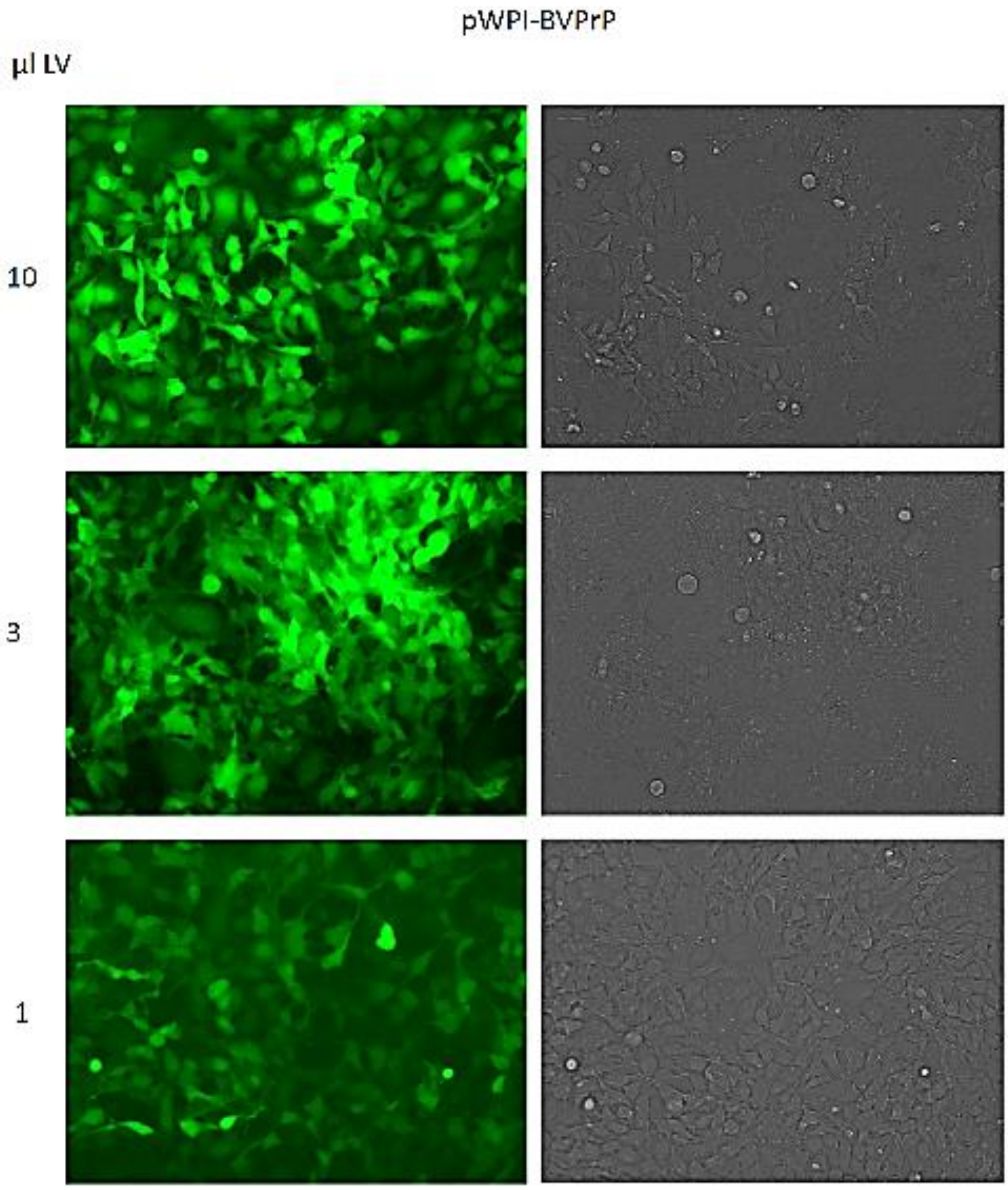

B

Supplementary Figure S3: Lentiviral titration in HEK293 cells. (A and B) Fluorescence and bright-field imaging of HEK293 cells transduced with pWPI-Cam-PrP expressing camel-PrP **(A)** and bank vole-PrP **(B)** lentivirus. GFP expression intensifies with increasing lentivirus from 1 to 10 µL, confirming efficient transduction, while some cell death is apparent at the highest dose.

Supplementary Figure 04

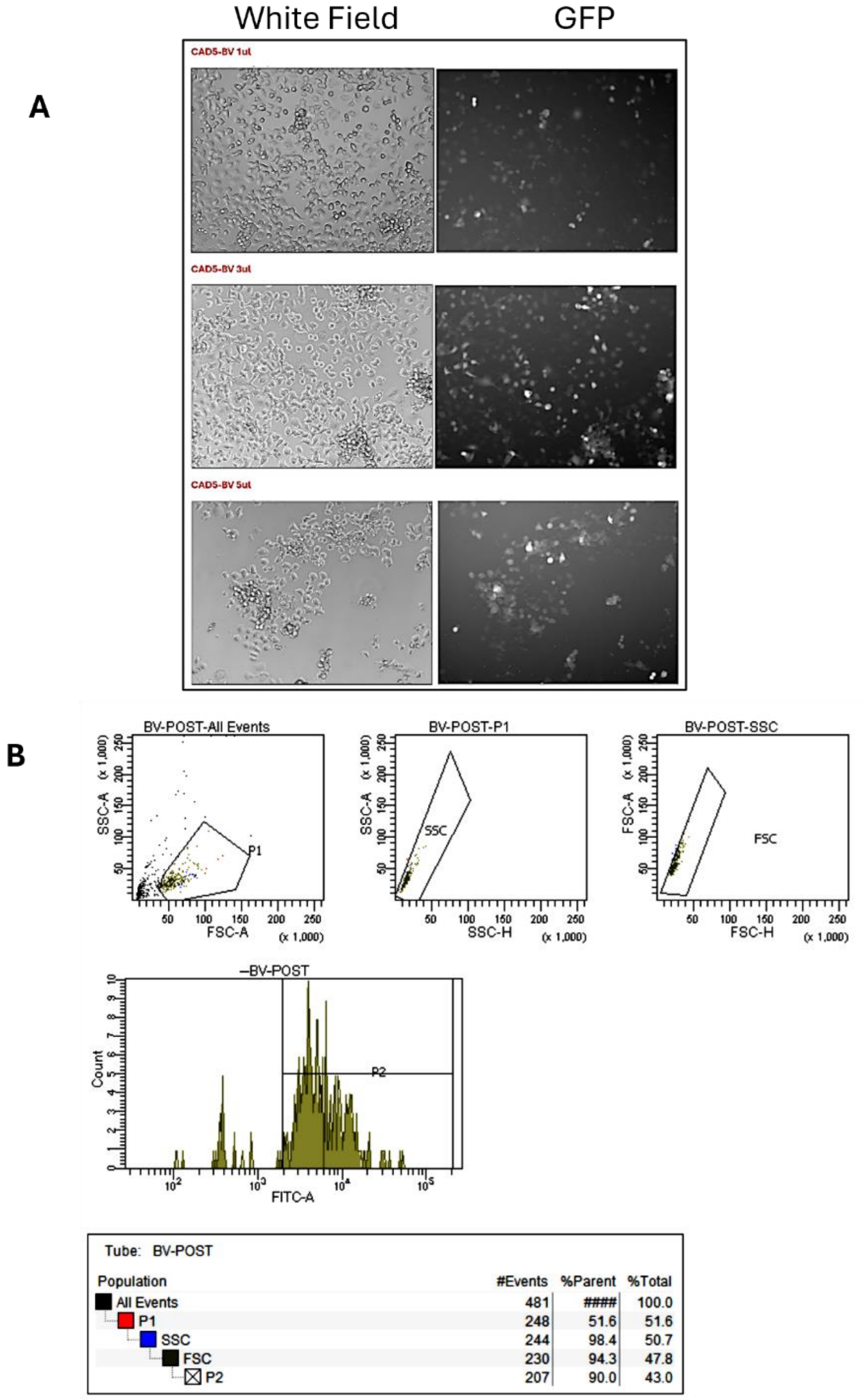

Tube: BV-POST

Population	#Events	%Parent	%Total
All Events	481	####	100.0
P1	248	51.6	51.6
SSC	244	98.4	50.7
FSC	230	94.3	47.8
P2	207	90.0	43.0

Supplementary Figure S4: Fluorescence imaging and FACS analysis of CAD5-bank vole-PrP transduced cells upon lentiviral transduction. (A) CAD5-PrP KO cells were transduced with 1 µL (top), 3 µL (middle), and 5 µL (bottom) of lentiviral particles carrying the bank vole-PrP gene. The left panels show bright-field images, while the right panels display the corresponding fluorescence images. Fluorescence intensity correlates with the volume of lentiviral particles used, reflecting the level of PrP expression. The 1 µL condition shows minimal and sparse eGFP fluorescence, indicating low transduction efficiency. The 3 µL condition yields increased eGFP expression with improved distribution across the cell population. The 5 µL condition demonstrated stronger fluorescence, in addition to a more uniform eGFP signal. **(B)** CAD5 cells expressing bank vole-PrP post lentiviral transduction. The top panels show scatter plots for all events, P1 gating, and SSC (side scatter) versus FSC (forward scatter), and histograms of GFP fluorescence intensity with the P2 gate indicating cells with high eGFP expression (middle panel). The tables summarize the number of events and eGFP statistics for each population.

Supplementary Figure 05

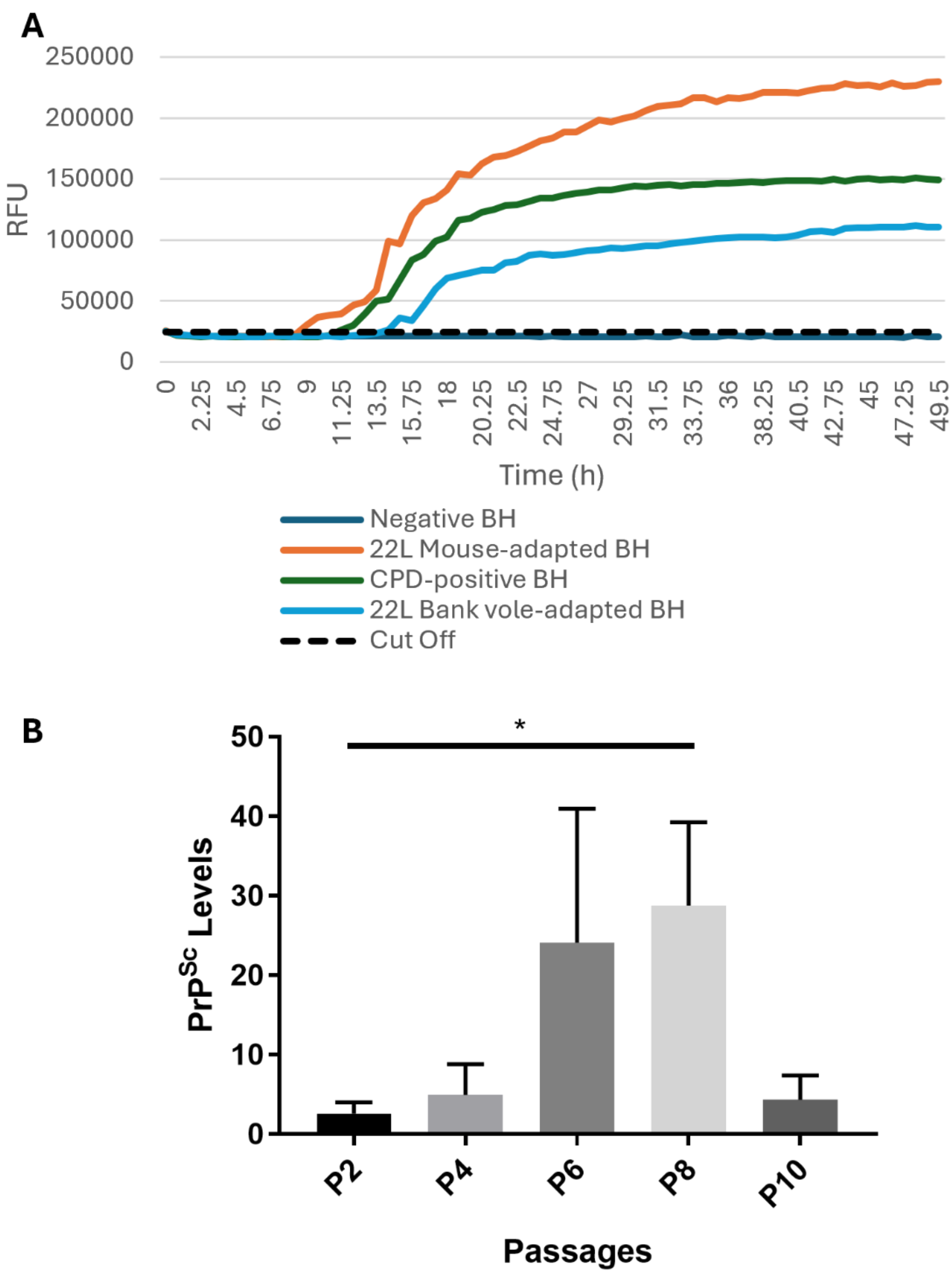

Supplementary Figure S5: RT-QuIC analysis of brain homogenates used in the infection experiment. (A) RT-QuIC for either uninfected naïve brain homogenate from C57BL/6 mice used as a negative control, 22L-infected mouse brain homogenate (C57BL/6), CPD-positive brain homogenate and 22L-infected bank vole brain homogenate. The average increase of thioflavin-T fluorescence of replicate wells is plotted as a function of time. The y-axis represents RFU, and the x-axis represents time (h). **(B)** Densitometric analysis representing the PrP^{Sc} signal of the immunoblot of Figure 3D (CAD5-Cam cells infected with CPrD brain homogenate). The data represent three different bio-replicates.

Supplementary Figure 06

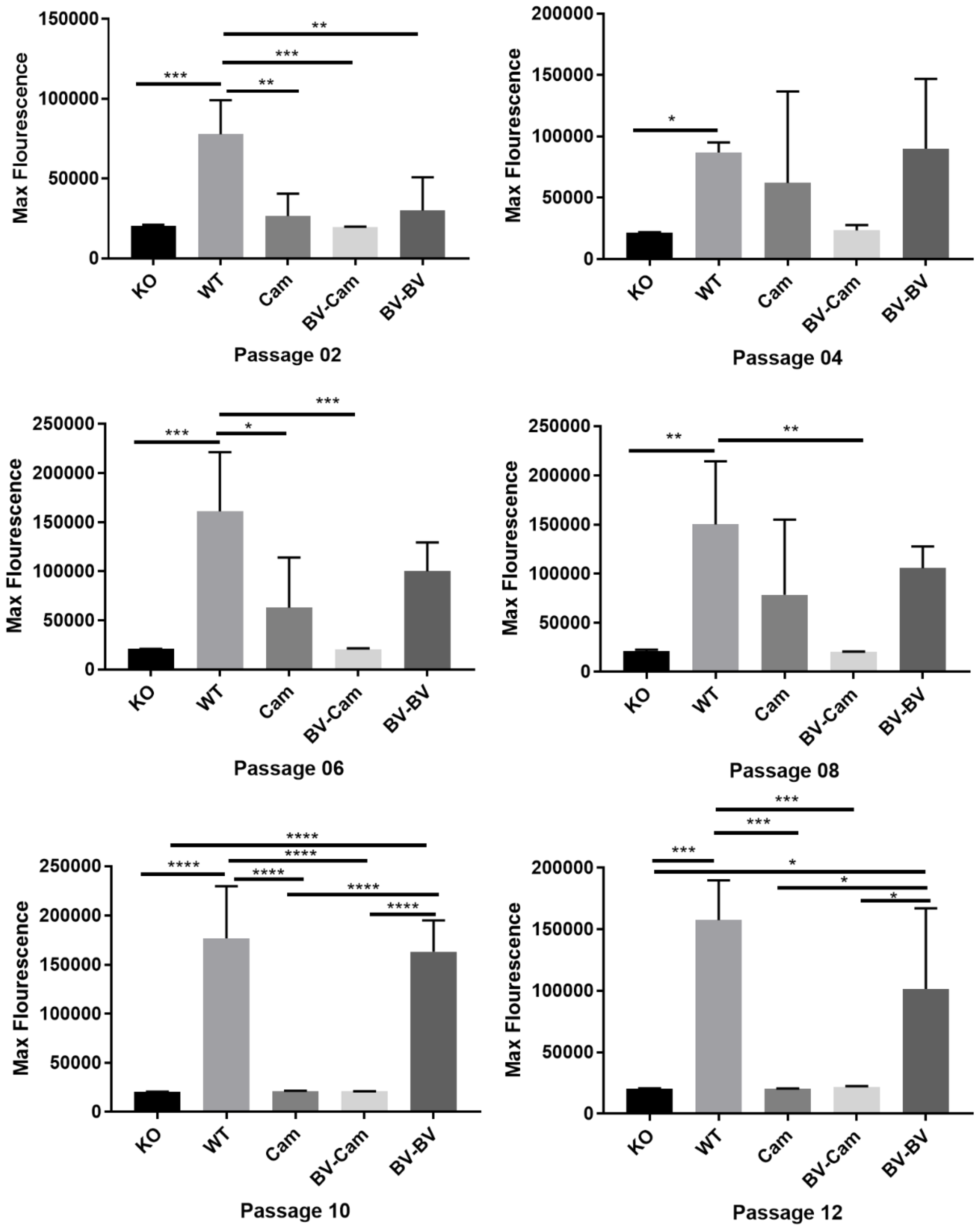

Supplementary Figure S6: Maximum fluorescence intensity for RT-QuIC analysis after infection. Statistical analysis of the maximum fluorescence intensity of RT-QuIC reactions after primary infection of CAD5 cells in passages 2 to 12. Statistical significance was expressed as follows: *, $p < 0.05$; **, $p < 0.01$; ***, $p < 0.001$; ****, $p < 0.0001$.

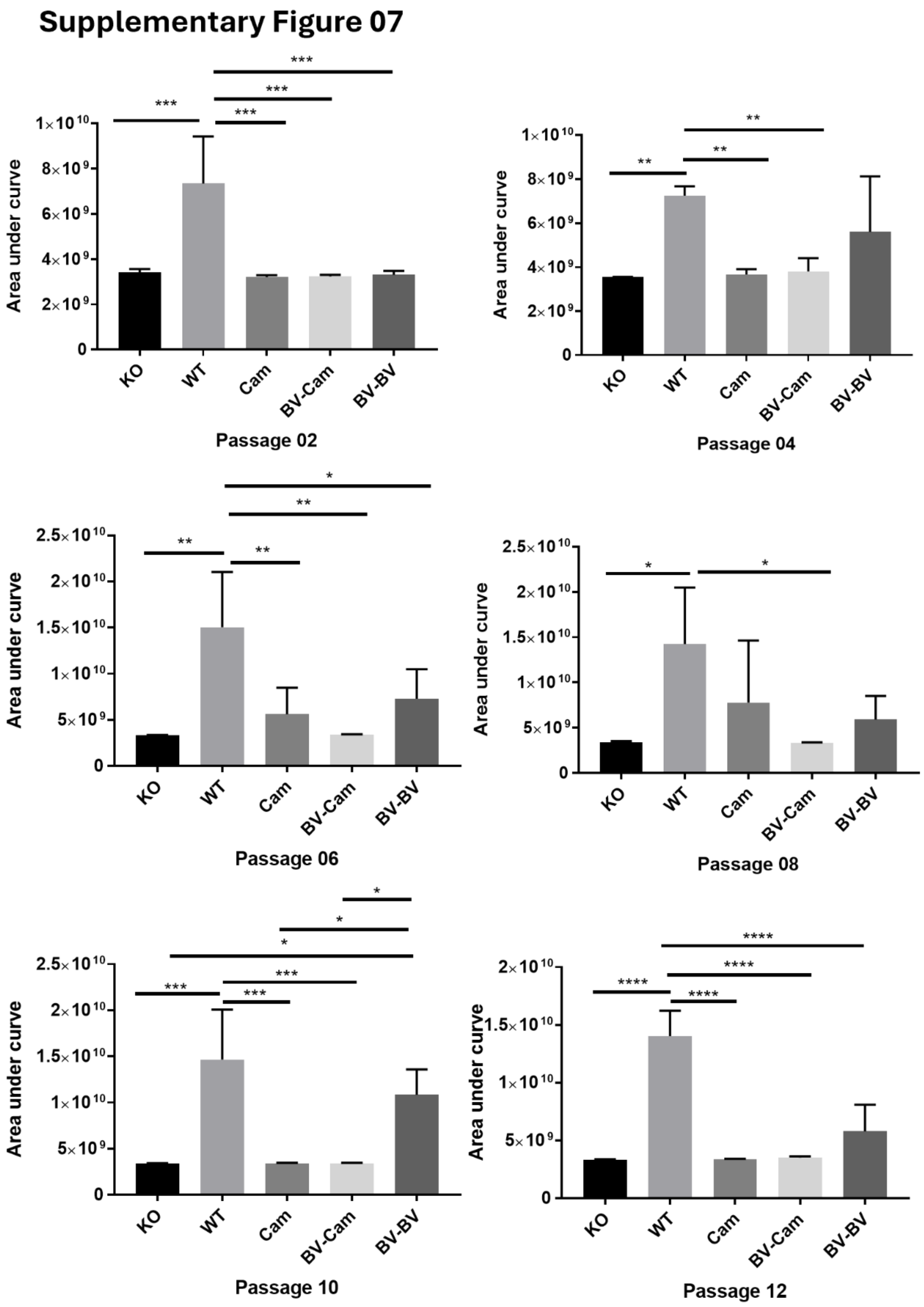

Supplementary Figure S7: The area under the curve for RT-QuIC analysis after infection. Statistical analysis of the area under the curve of RT-QuIC reaction after primary infection of CAD5 cells in passages 2 to 12. Statistical significance was expressed as follows: *, $p < 0.05$; **, $p < 0.01$; ***, $p < 0.001$; ****, $p < 0.0001$.

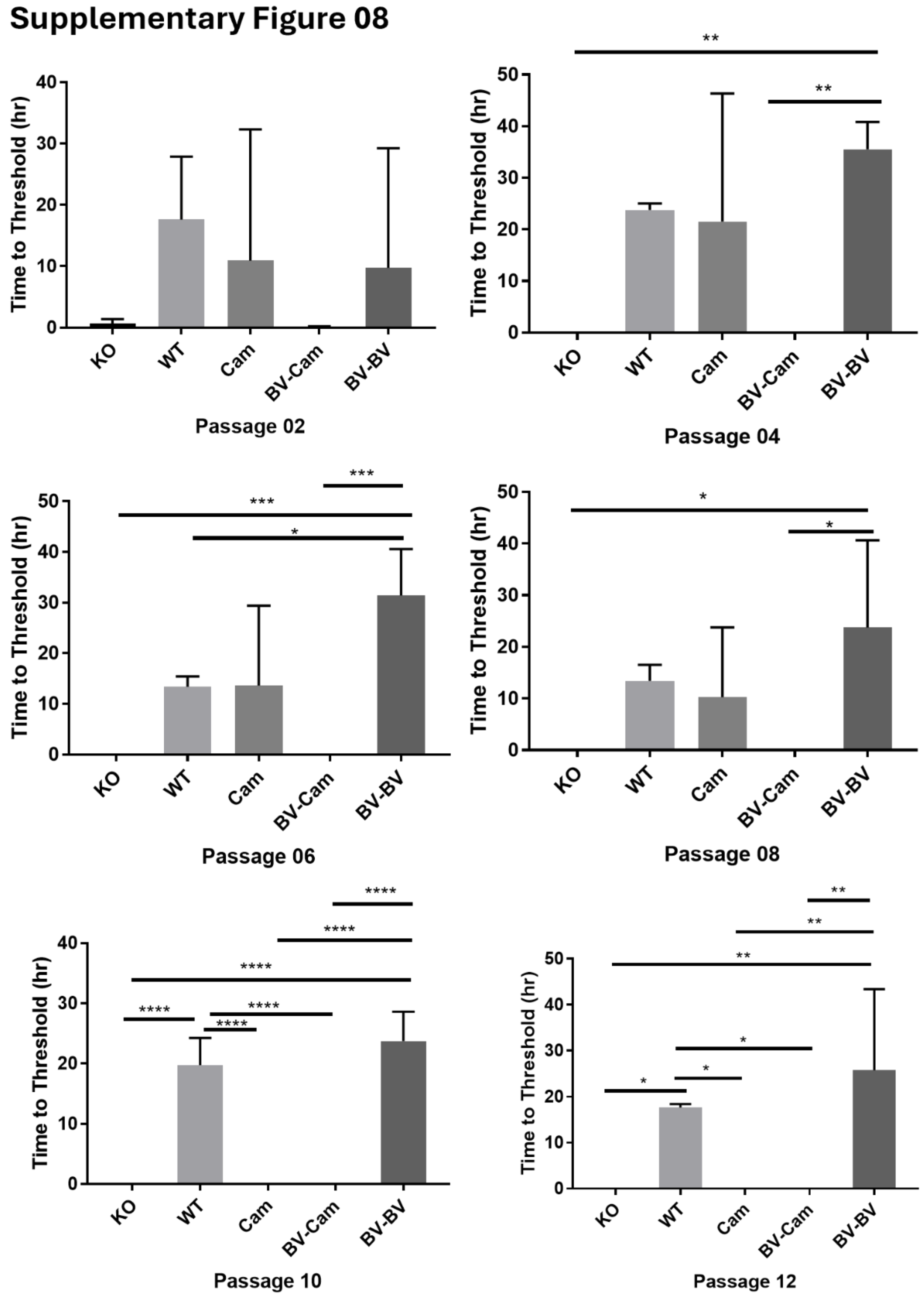

Supplementary Figure S8: Time to threshold values for RT-QuIC analysis after infection. Statistical analysis of the time to threshold values (hr) of RT-QuIC reactions after primary infection of CAD5 cells in passages 2 to 12. Statistical significance was expressed as follows: *, $p < 0.05$; **, $p < 0.01$; ***, $p < 0.001$; ****, $p < 0.0001$.
